# 

**Published:** 1911-12-02

**Authors:** 


*To the Editor of* The Hospital.

### Appointments.

The following appointments have been made at the Manchester Royal Infirmary: Mr. A. Ramsbottom, M.D., M.R.C.P., as Assistant Medical Officer (reappointed); Mr. T. T. Higgins, M.B., Ch.B. Vict., M.R.C.S., L.R.C.P., as Accident Room House Surgeon; Mr. J. B. Scott, M.B., Ch.B. Vict., and Mr. G. K. Thompson, M.B., Ch.B. Vict., House Physicians; Mr. J. R. Rigg, M.B., Ch.B. Vict., and Mr. W. A. Sneath, M.B., Ch.B. Vict., Senior House Surgeons; Mr. S. B. Radley, M.B., Ch.B. Vict., and Mr. W. H. Kauntze, M.B., Ch.B. Vict., Junior House Surgeons; Mr. F. H. Diggle, M.B., Ch.B. Vict., House Surgeon to Special Departments; Mr. Wm. J. Reid, M.A., B.Sc., M.B., Ch.B. Aberd., as Cancer Research Officer.

### Florence Nightingale Memorial.

WHY A STATUE OF FLORENCE NIGHTINGALE IS ESSENTIAL.

"The commemoration of great deeds and noble men and women by statues in public places is one of the best means of inculcating civic virtue. People see the statue, ask its history, and if they are worthy there quickens in them the desire to follow where the hero leads. Few women will look on a worthy statue of Florence Nightingale and not ask her story and learn some part of her lesson."—*Spectator*, Nov. 25, 1911.

We have received the following contributions since those acknowledged last week: Dreadnought Hospital, Greenwich (further subscription), £3 3s. 6d.; Miss C. Passmore, 5s. Received by Mr. G. Q. Roberts, Hon. Secretary: C. T. D. Crews, Esq., £100; Mrs. Craig Sellar, £100; Swansea General Hospital collection, £12 11s. 6d.; Sir Jeremiah Colman, Bart., £10 10s.; Mrs. Peyton, £10 10s.; G. W. Dawes, Esq., £10 10s.; Miss Frost, £10; Dowager Lady Ashburton, £10; Mrs. Clutton, £5 5s.; Geoffrey C. Cobb, Esq., £5 5s.; The Lady Wynford, £5; "In Memoriam St. Oliver Colt," £5; Fritz Reiss, Esq., £5; Hon. K. P. Bouverie, £3 3s.; John Tennant, Esq., £2 2s.; Mrs. Richard Pilkington, £2 2s.; A. N. Macnicoll, Esq., £2 2s.; Henry Wagner, Esq., £2 2s.; Miss J. G. Corbett, £2 2s.; S. H. Whitehead, Esq., £2 2s.; Lingholt School, Hindhead, £2 2s.; The Countess of Radnor, £2 2s.; D. B. Wilson, Esq., £2 2s.; Dr. and Mrs. Oliver, £2; and twenty-five sums of £1 or more. Further contributions may be sent to the Hon. Secretary, Florence Nightingale Memorial, St. Thomas's Hospital, Albert Embankment, London, S.E., or to the Editor of The Hospital, 29 Southampton Street, Strand, London, W.C.

### Gifts.

Mr. C. B. Hare, of Clifton, has left £1,000 to the Bristol General Hospital, £200 to the Royal Infirmary, and £100 to the Eye Hospital, Bristol.

Mr. Hugh A. Laird, of Blackheath Park, has left £1,000 to the Miller Hospital and Royal Kent Dispensary, £1,000 to the Scottish Hospital in London, and £1,000 to the Caledonian Asylum at Busbeg.

Mr. Thomas Layton, of Brentford, has bequeathed £100 each to the Female Lock Hospital, London, the Hospital for Diseases of the Throat, Golden Square, and the Cancer Hospital, Brompton.

Mr. Hy. Frederic Tiarks, of Chislehurst, has left £1,000 to the German Hospital, Dalston.

Under the will of Miss Margaret G. Lloyd-Roberts, of Ruthin, the Denbighshire Infirmary will receive a bequest amounting to over £5,000.

A sum of £240 has been sent anonymously to the Middlesex Hospital Cancer Charity.

Mr. William Hambidge, of Blackheath, has left £100 to the Blackheath and Charlton Cottage Hospital.

Sir Henry B. Samuelson, Bart., has sent a gift of pheasants for the patients of the Royal Hospital for Incurables, Putney Heath.

The Chesterfield and North Derbyshire Hospital has just received from the Duke of Devonshire, who is about to become Mayor of the borough, the sum of £100 from the Admission to Chatsworth Fund. It has also received from the estate of the late Mrs. Ann Waddington a legacy of £100.

Mrs. Emma Morrison, of Hastings, has bequeathed £500 to the Home for Little Boys at Swanley, and £200 to the Hastings Hospital. The residue of her property is left for the purpose of founding beds in hospitals to be named after her son, "George Edward Morrison Beds."

Mr. James Warnes Howlett, of Hove, has left £500 each to the Sussex Eye Hospital, the Royal Alexandra Hospital for Sick Children, Brighton, the Throat and Ear Hospital, Brighton, and the Sussex County Hospital. Also £500 to the Queen's Nurses, and £500 to the Dr. Barnardo's Homes.

